# Neurodiversity-affirming SEA for ADHD: evaluation of an online program for parents and children in the ultra-orthodox Jewish community

**DOI:** 10.1007/s00787-026-03063-x

**Published:** 2026-05-16

**Authors:** Haym Dayan, Mona Khoury, Eran Krivine, Yuval Bloch

**Affiliations:** 1 School of Social Work and Social Welfare, The Hebrew University of Jerusalem, Jerusalem, Israel; 2 School of Social Work, University of Haifa, Haifa, Israel; 3 The Hebrew University of Jerusalem, Jerusalem, Israel; 4 Shalvata Mental Health Center, Hod Hasharon, Israel; 5 Sackler Faculty of Medicine, Tel Aviv University, Tel Aviv, Israel

**Keywords:** ADHD, Salutogenic existential approach, Identity-affirming care, Self-determination theory, Culturally sensitive intervention, Ultra-Orthodox Jewish

## Abstract

In recent years, there has been growing recognition of the importance of affirmative interventions for neurodiverse individuals. However, empirical evidence regarding such interventions in ADHD remains limited, particularly within conservative-religious cultures. The present study examined the effectiveness of a culturally sensitive, online psychosocial intervention for parents and children. The intervention is grounded in a salutogenic existential approach (SEA), an integrative framework that conceptualizes ADHD as a tension between a tendency toward freedom and reality demands (necessity), and aims to enhance sense of coherence (SOC), autonomous motivation, and support for neurodivergent identity while integrating the medical model with the neurodiversity framework. The study included 427 participants from the Ultra-Orthodox Jewish (UOJ) community in Israel: 311 in the intervention group and 116 in the control group. The intervention group participated in a 12-session online program that combined weekly lectures and practice for parents, along with audio story episodes and a workbook for children. Assessments were conducted before and after the intervention or waiting period using parent-report questionnaires: ADHD symptoms and comorbid difficulties (VADPRS), child functioning (WFIRS-P), and parental self-efficacy (TOPSE). The intervention group demonstrated a significant reduction in ADHD symptoms, behavioral problems, and anxiety/depression, as well as significant improvements in child functioning and parental self-efficacy. In the control group, only a minimal reduction in ADHD symptoms was observed, with no changes in the other domains. Between-group differences remained significant after controlling for background variables. The findings indicate the potential of an SEA-based intervention to improve parent and child functioning among individuals with ADHD symptoms in the UOJ community, and highlight the contribution of SEA as an integrative model for addressing ADHD.

## Introduction

Attention-deficit/hyperactivity disorder (ADHD) is defined as a neurodevelopmental disorder characterized by inattention, impulsivity, and hyperactivity. The disorder is highly prevalent among children and adolescents worldwide [9] and in Israel [5], and is associated with adverse health, academic, and social outcomes [42].

ADHD commonly co-occurs with comorbid conditions such as behavioral disorders, anxiety, and depression [42], and is also associated with difficulties in parental functioning [111], including parenting self-efficacy, defined as a parent’s belief in their ability to successfully perform the parenting role [72]. Accordingly, a consistent association has been documented between parenting children with ADHD and lower levels of parental self-efficacy [15, 71, 90, 96, 113].

Recommended management of the disorder includes a combination of pharmacological treatment and psychosocial interventions [27, 115, 126]. Psychosocial interventions, including those delivered via the internet, may reduce ADHD symptoms and associated comorbid difficulties in children, and may also enhance parental self-efficacy [41, 58, 65].

Cultural [63] and religious factors [85] may influence the understanding of ADHD and coping strategies, underscoring the need to adapt intervention approaches to the target culture [25, 35, 130]. Culturally adapted psychosocial interventions have been shown to be feasible and effective [8, 60, 147].

However, there is a lack of evidence regarding psychosocial interventions tailored for ADHD in minority populations [95], including the Ultra-Orthodox Jewish (UOJ) community [21, 55]. The present study aims to address this gap.

The UOJ community in Israel constitutes approximately 13% of the population of the State of Israel [24]. This community is characterized by a collectivist social structure, strict adherence to religious commandments, obedience to authority, high demands for religious literacy, a conservative lifestyle, and reservations toward values perceived as modern [18, 54, 104]. Research on ADHD in the UOJ community in Israel offers a unique opportunity to examine a hidden population facing distinctive challenges at the individual, systemic, and clinical levels.

At the individual level, ADHD affects domains of functioning that are highly valued within Ultra- Orthodox culture, such as self-restraint, obedience, and intensive learning [21]. Failure to meet these expectations may be interpreted as religious or educational nonconformity, which may help explain reports of negative attitudes toward ADHD and toward the inclusion of diagnosed individuals within the community [105]. Consequently, youth with ADHD may perceive integration as unattainable and feel disconnected from their community [43].

At the systemic level, the literature highlights barriers to accessibility for diagnosis and treatment within the UOJ population [103]. For instance, lower rates of medical diagnosis acceptance and lower use of pharmacological treatment have been reported compared to the general population in Israel [67, 107]. Furthermore, UOJ patients have reported difficulties in communication with professionals regarding ADHD, noting that received recommendations often did not align with their cultural needs [23].

At the clinical level, cultural adaptation of interventions for the UOJ community requires consideration of cultural and religious values that shape attitudes toward mental health in general and toward ADHD in particular [109]. At present, two main approaches guide conceptualizations of ADHD - the medical approach and the neurodiversity approach -both of which present distinct challenges within the UOJ community.

1. **The medical approach**: this approach conceptualizes ADHD as a neurodevelopmental category resulting from deficits in brain functioning [135]. According to this approach, psychosocial intervention is deficit based and centers on symptom reduction and the enhancement of self-regulation, with the aim of facilitating the individual’s integration into society [28]. Within the UOJ community, the medical approach may be perceived as conflicting with the value of free will, according to which a “healthy” person is expected to be able to control their behavior [4]. As a result, this perspective may lead to denial of the objective difficulty or to the perception of ADHD as a moral or behavioral failure [85, 91, 124, 162], or alternatively to labeling individuals with ADHD as flawed or deviant within the community [99, 136].
2. **The neurodiversity approach**: this approach conceptualizes ADHD as a form of uniqueness that constitutes a legitimate part of human diversity, existing along a continuum and contributing to society. According to this perspective, a substantial portion of the difficulties associated with ADHD stems from a mismatch between the individual’s neurodivergent characteristics and the structure of society [133]. In line with this approach, psychosocial intervention is affirmative in nature and focuses on strengthening neurodivergent identity and adapting the environment to the individual, including the cultivation of a coherent neurodiversity identity, defined as a whole, consistent, and comprehensible sense of self; acceptance; an emphasis on strengths; the development of supportive environments; active involvement of the individual in the intervention; and the provision of coping tools for navigating the world as a minority [29, 56, 143, 160]. Within the UOJ community, the neurodiversity approach may be perceived as conflicting with the value of communality, according to which a “normal” individual is expected to conform to community standards [64, 75, 149, 150]. As a result, this perspective may lead to increased stigma or to the discouragement of recognizing ADHD as an objective phenomenon.

The challenge, therefore, is to provide neurodiversity-affirming intervention to individuals from the Ultra- Orthodox community, allowing space for a neurodivergent identity while maintaining community values. The implementation of the neurodiversity approach within the UOJ community serves as a case study relevant to other religious, conservative, or non-Western societies. Discussions surrounding neurodiversity paradigms have predominantly occurred within Western contexts [64].

The diversity paradigm touches upon Western values, such as individualism, and may not fully resonate within collectivist communities - whether East Asian, indigenous, or religious - where obedience, communal belonging, and traditional authority shape responses to neurodivergence [64, 97]. There is a growing need to examine the specific requirements of ethnically and culturally diverse populations within the neurodiversity framework [93, 102]. Furthermore, while evidence is accumulating regarding neurodiversity-affirming interventions for ADHD [19, 37, 122], research on such interventions within conservative-religious communities remains scarce. The present study seeks to address these gaps by evaluating the effectiveness of a culturally adapted, affirmative psychosocial intervention for ADHD in the Ultra- Orthodox community.

### Theoretical framework: SEA

The Salutogenic Existential Approach (SEA) is an approach to understanding and addressing ADHD that draws on empirically grounded principles and tools adapted from multiple theoretical frameworks. These include the salutogenic approach, which focuses on identifying sources of health such as sense of coherence; existential psychology, which addresses themes such as the tension between freedom and necessity; and self-determination theory, which emphasizes pathways to enhancing autonomous motivation [7, 30, 46, 120, 153].

SEA offers a complementary interpretive framework to the prevailing neuropsychological explanations, within which ADHD symptoms may be understood as an extreme manifestation of a mismatch between a tendency toward freedom and a reality that demands higher levels of structure (necessity). This mismatch increases vulnerability to symptoms as well as to emotional and functional impairment. Accordingly, the approach posits that interventions focused on addressing this tension are likely to result in improvements in both parent and child functioning.

The definitions of freedom and necessity presented below refer to a functional style rather than to ethical or political dimensions, or to personal needs or desires. The approach proposes a distinction between two functional styles: freedom, defined as a tendency to act as though reality is subordinate to the individual and the individual is not subject to constraints, and necessity, defined as a tendency to act as though the individual is subject to a reality governed by its own rules. The tension between these tendencies may be expressed in attentional style, with spontaneity-driven (exploratory, variable) versus task-directed attention; in modes of thinking, divergent versus convergent; in the perception of time and space, subjective versus objective; in emotional style, context-adjusted experience versus intense experience; in responsiveness to stimuli, restrained versus uninhibited; and in motor behavior, economical and goal-directed versus random.

The definition was formulated drawing on neuropsychological and psychoanalytic concepts. Functions described as “freedom” may be related to specific brain processes, such as the default mode network (DMN), which is associated with divergent thinking and mind wandering, characterized by inwardly focused attention and reduced attention to external stimuli, in contrast to the activity of task-positive networks (TPN), which are associated with attention to external stimuli and task-focused performance [94, 161]. In parallel, psychoanalytic conceptualizations describe the subjective tension between experiences of omnipotence, corresponding to freedom, and the recognition of and subordination to a limited reality, corresponding to necessity ([106, 154, 155], [156], [157, 158]).

Conceptualizing the disorder in terms of freedom and necessity enables the integration of an objective neurological explanation with a subjective psychological understanding [70, 77, 164]. This integration can be articulated through the three dimensions of sense of coherence proposed by the salutogenic approach: comprehensibility, a cognitive component referring to how the individual and their environment understand the disorder; meaningfulness, an emotional component referring to how the disorder is experienced and the meaning attributed to it; and manageability, a behavioral component referring to how the individual and their environment perceive their capacity to cope with the disorder.

**Comprehensibility**. SEA proposes that ADHD symptoms can be understood as an expression of an extreme tendency toward freedom over necessity, meaning that the individual’s inner world is conceptualized as manifesting patterns of freedom rather than necessity across functions related to attentional style, volition, and movement. This conceptualization is consistent with the Default Mode Interference Hypothesis, according to which ADHD may involve specific patterns of interaction between the default mode network and task-directed networks, at times reflected in reduced suppression of default mode network activity during task demands [47, 86, 134, 139].

This description integrates *explanation*, an objective “outside” perspective on functioning emphasized by the medical approach, with *understanding*, a subjective “inside” perspective on how individuals perceive themselves, as reflected in the neurodiversity approach. The description focuses on functioning rather than on choice, such that ADHD symptoms are not viewed as behaviors freely chosen by the individual. At the same time, it allows for an affirmative understanding of the person with ADHD, emphasizing who the person is rather than only who they are not in comparison to others [17] .

To apply this explanatory and interpretive framework in practice and to create a shared, accessible language [12, 140], the approach introduces the metaphors of the “circle”, representing a tendency toward freedom, and the “square”, representing a tendency toward necessity. These metaphors were inspired by Leonardo da Vinci’s Vitruvian Man [49]. Accordingly, domains of impact of the disorder are divided into subdomains presented along the freedom-necessity continuum, for example, “square attention”, characterized by focus and systematic processing with the exclusion of distractions or stimuli irrelevant to the defined task, versus “circular attention”, characterized by an exploratory style that moves across topics with a low threshold for curiosity [138]. These metaphors make it possible both to acknowledge the distinctive qualities of the “circle” (freedom) and to appreciate the importance of the “square” (necessity) for adaptive functioning in the external environment, thereby granting recognition and space to both dimensions while directing attention to context, namely, when and to what extent circular or square functioning is required.

**Meaning**. The way in which the disorder is experienced is influenced by on the social and personal meaning attributed to it. Conceptualizing the trait underlying the disorder as a tendency toward freedom involves assigning meaning to freedom as a distinct and valuable mode of functioning, rather than as a absence of adaptation to environmental demands. This conceptualization enables a shift from a dichotomous discourse, framing ADHD either as an “impairment with risk potential”, as emphasized by the medical approach, or as a “valued difference with advantages”, as proposed by the neurodiversity approach, toward a dialectical discourse that understands the tendency toward freedom as contextual potential, encompassing both risks and opportunities, advantages and disadvantages. Within this framework, there are contexts in which a tendency toward freedom may support the individual and contexts in which it may be detrimental; difficulties may arise from a mismatch with modifiable structural demands, such as homework, or from a mismatch with rigid structural demands, such as refraining from disturbing others [11, 31, 62, 82, 133].

This meaning is accompanied by an emotional-experiential dimension. At this level - beyond freedom as a functional style - the tension between freedom and necessity can also be understood as a lived experience. Rather than focusing solely on the secondary emotional aspects of the disorder, such as shame or disappointment associated with functional difficulties, attention can also be directed to the emotional dimension arising from this tension itself. Just as anxiety has been conceptualized as an intensification of natural vigilance [1], ADHD may be understood as an intensification of a natural human tension between freedom and necessity - a tension experienced by all people, for example through feelings of relief when released from coercion or in unstructured activity [52]. This framing supports the humanization and empathic understanding of the individual with the disorder, alongside recognition of both the positive experiential meaning inherent in the tendency toward freedom and the suffering that may arise when this experience is not subject to voluntary control. It further enables individuals to choose a personal narrative that provides meaning and self-worth, while distinguishing between scientific facts and value-based meanings that remain a matter of personal choice [20, 92, 110, 163]. For example, pharmacological treatment may be perceived as a tool used by the individual to temporarily strengthen the “square” capacity, rather than as passive treatment or as evidence of a defect.

**Manageability**. The perceived ability of an individual with ADHD to function within their environment is closely related to how coping with the disorder is conceptualized. Both the medical approach and the neurodiversity approach aim to promote change, whether in the individual or in the environment, in order to reduce the gap between the individual’s tendency toward freedom and the necessity of the environment. SEA proposes a third pathway: recognizing and accepting this tension as an existential reality of life experienced by the general population, and in a more pronounced manner by individuals with ADHD. Rather than attempting to eliminate the tension, the focus is on changing one’s relationship to it and actively managing it. This may involve distinguishing between life domains in which a tendency toward freedom creates difficulties and those in which it is beneficial, or identifying contexts in which the individual naturally succeeds in managing the tension between freedom and necessity, thereby strengthening the sense of having resources to do so. This approach also encourages examining what works for others who cope successfully, reflecting the salutogenic principle of learning from health [6].

This framework - conceptualizing the disorder as a tendency toward freedom, understanding freedom as a human expression with contextual potential, and viewing the freedom-necessity tension as manageable - guides the therapeutic intervention. In contexts in which a tendency toward freedom creates tension and a mismatch with environments, whether physical, psychological, or social, that require higher levels of structure (necessity), SEA proposes managing this tension through psychological mechanisms that operate naturally across the general population. These mechanisms reshape the individual’s perception of freedom and necessity and promote improved functioning and mental health. Such mechanisms are articulated within the salutogenic approach and self-determination theory, and SEA proposes strengthening them through targeted intervention for ADHD.

**The salutogenic approach** - this approach proposes that the tension an individual experiences between their tendencies and the demands of reality may elicit two possible responses: a sense of chaos, characterized by confusion, emptiness, and helplessness, or a sense of coherence, comprising comprehensibility, meaningfulness, and manageability [7, 148]. Previous studies suggest that coping with ADHD is associated with experiences of chaos, disorder, and lack of control [51, 144]. In addition, a negative association has been found between ADHD symptoms and sense of coherence among individuals with the disorder as well as among their parents [39, 66, 98]. Higher levels of sense of coherence have been linked to fewer ADHD-related functional impairments [2, 33, 34]. Importantly, sense of coherence has been shown to be amenable to improvement through therapeutic intervention [81, 87]. The salutogenic approach has been identified in the literature as relevant to advancing discourse on neurodiversity [45], and the SEA intervention draws on its foundations with the aim of enhancing sense of coherence in coping with ADHD.

**Self-determination theory (SDT)** - the - theory proposes that engagement with reality and the need to function within it may lead either to controlled motivation, experienced as coerced action driven by external constraints, or to autonomous motivation, characterized by acting out of interest, enjoyment, or an understanding of the importance and value of the activity [120]. Research has shown that autonomous motivation is associated with psychological growth, mental well-being, and optimal functioning [119]. According to the theory, autonomous and controlled motivation differ in their degree of internalization, defined as the process through which external demands such as norms and rules are integrated into the self, such that individuals experience them as reflecting who they are or what they genuinely want. Internalization is facilitated through the satisfaction of three basic psychological needs: autonomy, the need to experience behavior as self-endorsed and freely chosen; relatedness, the need to feel connected to family and community; and competence, the need to feel capable of realizing one’s internal goals within external constraints.

Self-determination theory has been identified as relevant to ADHD, in part based on findings indicating that reduced motivation predicts impairments in ADHD-related functioning [57, 101, 131]. Individuals with ADHD have been found to experience reduced satisfaction of basic psychological needs, a deficit that mediates decreased motivation [36, 108, 118, 123]. For example, children with ADHD tend to act more under pressure from external stimuli and exhibit higher levels of amotivation [26, 129]. In contrast, autonomous motivation has been shown to predict academic achievement and improved concentration [117, 132, 146]. Similarly, autonomy support has been identified in qualitative studies as a facilitating factor and has been associated with improved functioning and increased sense of coherence among individuals with ADHD [44, 88, 100, 127, 128, 141]. Interventions designed to support autonomous motivation have been shown to be feasible and effective [74, 89]. Accordingly, SEA draws on core principles of self-determination theory with the aim of enhancing support for basic psychological needs in the context of ADHD.

### Intervention framework

SEA serves as a framework for a psychosocial intervention through a structured protocol consisting of lectures, experiential storytelling, and CBT-based practice. The intervention is based on iterative application of a unified set of tools across multiple functional domains relevant to ADHD. Within each domain, two functional poles, freedom and necessity (represented by the “circle” and the “square”), were introduced, and participants practiced three core components.

The SEA originality stems from its focus on expanding the capacity for free choice, rather than compliance. It achieves this approach by working simultaneously on two axes: a perceptual-identity axis, enhancing comprehensibility, meaning, and manageability; and a motivational axis, supporting autonomy, relatedness, and competence. Critically, intervention begins from the “circle” - validating and strengthening the freedom orientation - and moves toward the “square” from within it, rather than imposing structure from without. The focus of change is neither the child nor the environment but the integration between them.

The *cognitive component* focused on developing understanding, awareness, empathy, and a shared language between parents and children. This included reframing ADHD manifestations through the metaphors of the “circle” and the “square”, which recognize these tendencies as universal and valuable; and encouraging parents to support autonomy.

The *emotional component* focused on supporting neurodivergent identity through meaning, positive narratives, compassion, and acceptance. This involved identifying inhibiting and supportive meanings attributed to the disorder and coping with it; offering positive meanings to reduce negative stigma; validating freedom as a universal human experience; emphasizing intrinsic motivation for developing regulatory skills; promoting self-advocacy by children and parents; and strengthening a sense of belonging within the family and community. For instance, parents were guided to identify “circular” behaviors in the child that are inherently charming or possess developmental potential, and to recognize these traits as possessing intrinsic and purposeful value.

The *behavioral component* aimed to enhance manageability and competence by focusing on managing the tension between freedom and necessity rather than attempting to eliminate it. This included identifying contexts in which a tendency toward freedom is beneficial and reinforcing them; identifying situations in which individuals already succeed in balancing freedom and necessity and transferring these coping patterns to new contexts; setting graded goals for parents and children; and providing tools to strengthen parental authority and connection [80, 112, 145].

Repeated application of the same set of tools across varying functional domains was intended to promote internalization and generalization to daily life routines. For example, everyday situations were analyzed according to three components: what the child wants (comprehensibility), why it is important to them (meaningfulness), and how they cope in practice (manageability). Specifically, parents practiced analyzing daily conflicts through a dual lens: the “Square” perspective - focusing on environmental requirements - and the “Circular” perspective - examining how the child perceives the situation. Parenting practices emphasized three forms of “nourishment”: boundaries and goal setting to support competence; freedom and preservation of uniqueness to support autonomy; and negotiation, flexibility, and compromise to support relatedness.

#### Cultural adaptation

The proposed framework enables integration of the medical and neurodiversity approaches by accommodating neurological aspects alongside existential and experiential dimensions, thereby mitigating conflict with community values. The intervention also included culturally specific elements, such as addressing religious functions affected by ADHD - for example, explaining prayer through the circle-square framework as involving both self-expression and communal structure, and guiding parents to connect the child to prayer through their personal style. It further linked acceptance of difference to community values by presenting neurodiversity through sources that praise God for creating a diversity of human styles, thereby translating this idea into a familiar religious language, and framing the concept of “freedom” as a foundation for “liberty”, a concept with deep historical significance in Jewish tradition (Josephus, [73]).

#### Study goals and hypotheses

To date, no published studies have examined an ADHD intervention that integrates the three components of sense of coherence, the three basic psychological needs, and neurodiversity-affirming support specific to ADHD as primary intervention targets. Nevertheless, the literature reviewed above points to the potential of integrating these components within a culturally sensitive framework to improve parent and child functioning. Accordingly, the aim of the study was to examine changes in parent and child functioning following a parental skill-based intervention, compared with a comparison group. The study hypothesized that participation in the intervention would be associated with improvements in primary outcomes directly targeted by the program: (a) parent functioning, reflected in increased parental self-efficacy and (b) child functioning, including reductions in ADHD symptoms and behavioral problems. Secondary analyses explored whether intervention participation was additionally associated with changes in child anxiety, depression, self-concept, social activities, risky behaviors, and improvements in family functioning and life skills.

Accordingly, the aim of the study was to examine changes in parent and child functioning following the intervention, compared with a control group. The study hypothesized that participation in the intervention would be associated with improvements in primary outcomes directly targeted by the program: *(a)* child functioning, including reductions in ADHD symptoms and behavioral problems; and *(b)* parent functioning, reflected in increased parental self-efficacy. Secondary analyses explored whether intervention participation was additionally associated with changes in child anxiety, depression, self-concept, social activities, and risky behaviors.

## Methods

### Participants

Participants were 361 families recruited through notices on bulletin boards in local UOJ municipalities, press releases, mailing lists, and word of mouth. Recruitment occurred between November 2023 and March 2024, targeting parents of children aged 6–16 years diagnosed with ADHD by community doctors. Due to the challenges in obtaining proper medical evaluations within this specific community, we also included parents of children suspected of having ADHD but not formally diagnosed (62% had a formal ADHD diagnosis). Participants were informed that the intervention was intended as a psychosocial supplement to standard care and was not a substitute for professional diagnosis or pharmacological treatment when warranted. The aim of the intervention was to enhance adherence to medical care rather than replace it.

**Fig. 1:**
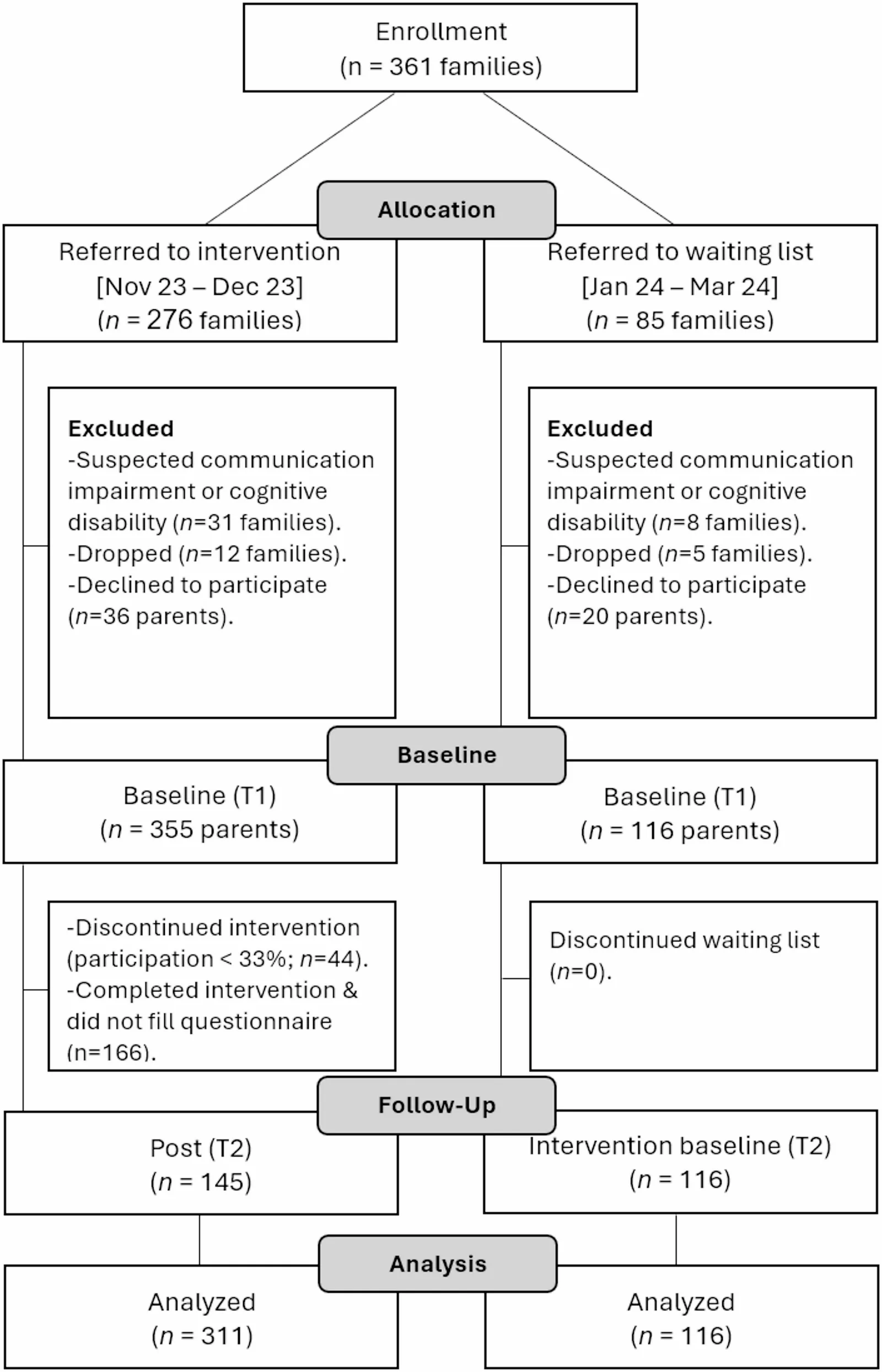
CONSORT diagram showing the participants’ flow through each stage of the research and reasons for dropout

As described in Fig. 1, families who were recruited until December 2023 (*n* = 276) were allocated to the intervention group and those who were recruited from January to March 2024 (*n* = 85) were referred to the waiting list group. From the intervention group, 79 families were excluded due to suspected cognitive impairment or communication disorder (*n* = 31), or because an individual rather than group intervention was deemed necessary, drooped out (*n* = 12) or declined participation (*n* = 36). Of the potential 430 parents, 355 (89%) parents completed the baseline measurement. Of these, 44 (12%) discontinued the intervention after participating in less than 3 meetings. Thus, the intervention group consisted of 311 parents. For the comparison group (waiting list), 33 families were excluded due to suspected communication or cognitive impairment (*n* = 8), dropped out (*n* = 5), or declined participation (*n* = 20). Of the potential 124 parents, 116 (94%) completed the baseline measurement. None of the parents dropped during the waiting list. Analysis of dropout rates revealed no statistically significant differences in socio-demographic variables between participants who dropped out and those who continued with the intervention. To determine power, we used G*power with f = 0.25 (i.e., small effect size) and α < 0.05. For the current sample size, power was approximately 1.

### Intervention

The Intervention led by a PhD social worker from the UOJ community was developed in consultation with community leaders to ensure alignment with UOJ values. The therapeutic team included clinicians who identify as having ADHD themselves. This aspect of the team composition aligns with contemporary literature on neurodiversity in clinical settings and is consistent with inclusive practices that value lived experience as an important professional asset [61].

Funding was provided by various public authorities, and a nominal participation fee was either charged or waived, depending on the policy of the respective authority.

The intervention was delivered concurrently to all participants. Parents attended 12 one-hour sessions via video or telephone. At the end of each session, they received weekly practice guidelines, and a question-and-answer segment was provided, lasting approximately 45 min on average. Parents also received session summaries and practice materials via email, along with access to session recordings either through the program’s website or a voice-based platform (a telephone-based alternative to the internet designed for the UOJ community). Children were given access to an 11-episode psychoeducational audio story, with each episode averaging 10 min and focusing on life skills relevant to coping with ADHD. The audio content was accompanied by a workbook. Parents received reminders via email or phone close to the time of each session. Attendance was recorded based on family logins to the voice-based platform or the program website, and ranged from 5 to 12 sessions, with a mean of 10.32 ± 1.70.

### Measures

**Vanderbilt ADHD.** The Vanderbilt ADHD Diagnostic Parent Rating Scale (VADPRS) is a 55-item self-report scale for parents assessing ADHD and comorbid conditions across six different domains: Inattention, Hyperactivity/Impulsivity, Oppositional Defiant Disorder, Conduct Disorder, Anxiety/Depression and Performance [159]. Each item from the symptoms scales is rated on a 4-point Likert scale ranging from 0 (never) to 3 (very often). The performance scale is rated on a 5-point Likert scale ranging from 1 (excellent) to 5 (problematic). In accordance with previous studies, in the present study, we calculated a total measure of ADHD symptom severity (Inattentive and hyperactive-impulse domains). Cronbach’s alpha values were high for all the VADPRS dimensions: ADHD (pre 0.88, post 0.89), oppositional defiant disorder (pre 0.87, post 0.90), conduct disorder (pre 0.88, post 0.91), anxiety/depression (pre 0.85, post 0.86), and performance (pre 0.71, post 0.75).

**Weiss Functional Impairment Rating Scale (WFIRS).** The WFIRS is a validated self-report tool designed to assess functional impairment in individuals with Attention-Deficit/Hyperactivity Disorder (ADHD) across multiple life domains [142]. It includes 50 items divided into six subscales: Family, School/Work, Life Skills, Self-Concept, Social Activities, and Risky Activities. Parents rated each item on a 4-point Likert scale ranging from 0 (never or not at all) to 3 (very often or very much). A total score was derived by averaging all items, with higher scores indicating greater functional impairment. Cronbach’s alpha reliability was high at baseline 0.92 and at the post intervention 0.94.

**Parenting Self-Efficacy.** Parenting self-efficacy was measured with the well-validated 48-item TOPSE [76]. Participants responded to each statement on a Likert scale ranging from 0 (completely disagree) to 10 (completely agree). The TOPSE score was calculated as the mean of all 48 items, with higher scores reflecting greater confidence in parenting abilities. The instrument exhibited strong internal consistency, with α = 0.94 prior to the program and 0.95 afterward.

#### Demographic Questionnaire

The questionnaire collected demographic information about the parent and the child, including age, gender, income level (Likert scale ranging from 1(very low) to 5 (very high)), employment status (employed, studying in religious seminaries called yeshivot or kollelim, unemployed), and education level (elementary, secondary, post-secondary including yeshiva, and academic).

### Data analyses

Data analyses were conducted by using SPSS V.29). Descriptive statistics for the study’s variables were examine, also an independent samples t-test for the continuous research variables and a chi-square test for the categorical variables were employed to test differences between the intervention and comparison groups before the study. To test the research hypotheses, a repeated-measure ANOVA was used with the pre-post intervention assessment as a within factor and group (intervention vs. comparison) as a between factor. In all analyses, alpha was set to 0.05 and corrected for multiple comparisons using the Benjamini-Hochberg adjustment [14]. Effect sizes are represented by partial eta squared. Missing data were handled using the maximum likelihood approach. For robustness of analyses, we additionally provide a listwise approach to missingness in the supplemental materials. To test for deviation from normality, we used the Skewness > 2 and Kurtosis > 7 criteria [59]. Except for conduct disorder, all variables did not show deviation from normality (see Table 1 s in the supplemental materials).

## Results

Table 1 presents the socio-demographic characteristics of participants in the intervention and control groups. Except for minor significant differences, the intervention and comparison groups were similar in their socio-demographic characteristics. Parents in the intervention group had children who were about one year younger, had slightly more children, lower income, and fewer were employed. The groups did not differ in child and parent functioning variables, including the proportion of children with a formal ADHD diagnosis.

**Table 1:** Descriptive statistics of the intervention and control groups

	Intervention (*n* = 311)			Control (*n* = 116)			
	M	(SD)		M	(SD)	t value	*p*
Child age	9.45	(2.01)		10.46	(2.02)	4.57	< 0.001
Father age	37.64	(5.99)		38.73	(5.54)	1.71	0.088
Mother age	35.94	(5.48)		37.45	(5.09)	2.58	0.010
No. of children	5.96	(2.07)		5.37	(2.12)	2.57	0.011
Birth order	2.77	(2.16)		2.62	(1.81)	0.66	0.512
Age at diagnosis	7.70	(1.86)		8.06	(1.88)	1.46	0.145
Income	2.84	(0.62)		2.99	(0.58)	2.34	0.019
	*%*	*(n)*		*%*	*(n)*	*χ* ^*2*^	*p*
Child gender (boys)	68%	(210)		63%	(73)	0.80	0.372
Formal ADHD diagnosis	61%	(190)		65%	(76)	0.70	0.401
Parent gender (females)	55%	(170)		53%	(54)	0.05	0.823
Participating parent (both)	88%	(273)		84%	(98)	0.81	0.369
Education type (regular class)	98%	(306)		96%	(111)	2.70	0.100
Marital status (married)	99%	(309)		97%	(113)	4.65	0.031
Occupation							
Working	61%	(191)		74%	(86)	6.14	0.046
Studying	31%	(96)		22%	(25)		
Not working	8%	(24)		4%	(5)		
Parent education							
Secondary or less	7%	(17)		7%	(6)	0.31	0.858
Post-secondary	67%	(154)		64%	(54)		
Academic	26%	(61)		29%	25)		

**Fig. 2:**
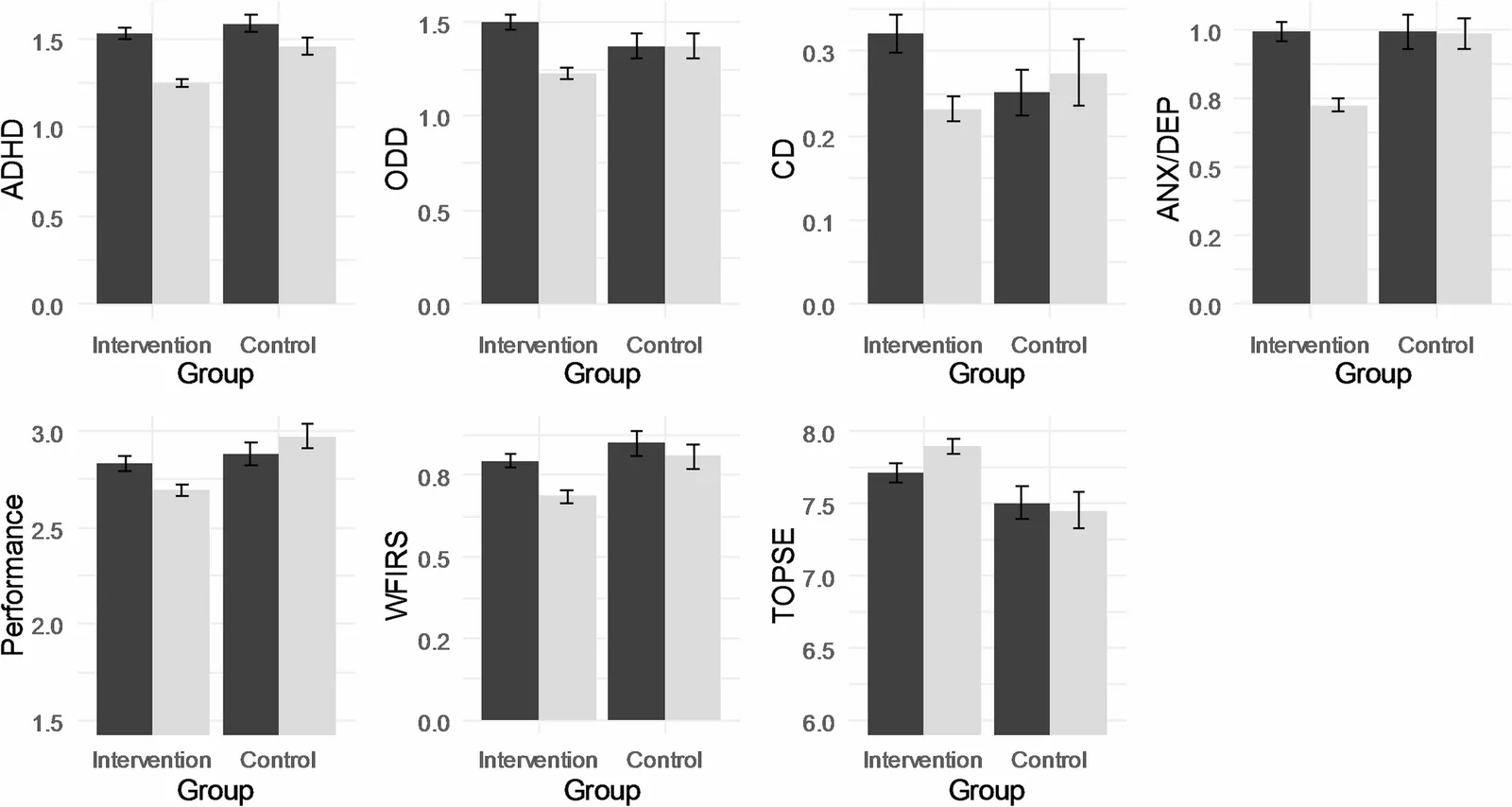
Pre to Post changes in all the research variables

As presented in Table 2, results of a two-way repeated measures ANOVA showed consistent and significant group by time interaction effects for all the research variables. Probing the interactions, as presented in Fig. 2, revealed that the intervention group did not significantly differ from the control group at baseline in all variables (ADHD *p* =.475; ODD *p* =.131; CD *p* =.119; distress *p* =.994; performance *p* =.475; TOPSE *p* =.118; WFIRS *p* =.254). Participants in the intervention group reported a significant decrease in child ADHD and comorbid dimensions (ADHD *p* <.001; ODD *p* <.001; CD *p* <.001; distress *p* <.001), decrease in child impairment functioning (Vanderbilt performance *p* <.001; WFIRS *p* <.001), and increase in parental efficacy (TOPSE *p* <.001). In contrast, participants in the control group reported a smaller decrease in ADHD (*p* =.002) but did not show a significant change in the other comorbid dimensions (ODD *p* =.984; CD *p* =.468; distress *p* =.914), nor in the child impairment functioning (Vanderbilt performance *p* =.068; WFIRS *p* =.084), and parental efficacy (*p* =.441).

**Table 2:** Pre-Post comparisons of the research groups

	Pre	Post		Time			Group			Time × Group	
	*M *±* SD*	*M ± SD*	*F*	*p*	*η*_*p*_^*2*^	*F*	*p*	*η*_*p*_^*2*^	*F*	*p*	*η*_*p*_^*2*^
ADHD											
Intervention	1.52 ± 0.60	1.25 ± 0.44	6.47	<.001	.13	3.14	.026	.02	7.17	.007	.02
Control	1.59 ± 0.51	1.46 ± 0.54									
ODD											
Intervention	1.49 ± 0.71	1.23 ± 0.55	17.15	<.001	.04	0.04	.842	.00	17.44	<.001	.04
Control	1.37 ± 0.67	1.37 ± 0.68									
CD											
Intervention	0.32 ± 0.41	0.23 ± 0.27	2.60	.135	.01	0.11	.743	.00	8.14	00.7	.02
Control	0.25 ± 0.30	0.27 ± 0.42									
Anx/Dep											
Intervention	0.99 ± 0.69	0.73 ± 0.48	24.43	<.001	.05	4.31	.055	.01	22.65	<.001	.05
Control	0.99 ± 0.67	0.99 ± 0.61

We conducted additional analyses to ensure the robustness of our findings. First, we re-ran the repeated measures ANOVA while controlling for child age, number of children in the family, family income, and parental occupation - variables that differed between the intervention and control groups. The results showed that the group-by-time interactions remained significant across all research variables, suggesting that socio-demographic differences between groups do not account for the intervention’s effectiveness (see Table 2 s in the supplemental materials). Second, to ensure that the results were not biased by the maximum likelihood approach for handling missing data, we replicated the analyses using the traditional listwise deletion method in the repeated measures ANOVA routine. The results indicated that the group-by-time interactions remained significant across all research variables, suggesting that the method of handling missing data did not bias the findings (see Table 3 s in the supplemental materials). Third, since conduct disorder scores were not normally distributed, we assessed the intervention’s effect using the Wilcoxon non-parametric test. Consistent with the repeated measures ANOVA, the Wilcoxon test revealed a significant reduction in conduct symptoms in the intervention group (*Z* = 5.51, *p* <.001) but not in the control group (*Z* = 0.34, *p* =.732). Finally, to address potential concerns regarding diagnostic heterogeneity, we conducted a replication analysis restricted to children with a formal ADHD diagnosis. Results revealed significant group × time interaction effects for five of the seven dependent variables, with marginally significant effects observed for conduct symptoms and the WFIRS (see Supplemental Table 4), thereby corroborating the primary findings.

## Discussion

The present study examined the effectiveness of a culturally sensitive, affirmative psychosocial intervention for addressing ADHD symptoms among parents and children from the UOJ community. The findings indicate significant improvements among participants in the intervention group in child functioning, alongside improvements in parent functioning reflected in increased parental self-efficacy. In contrast, no significant changes were observed in the comparison group, with the exception of a relatively small but significant reduction in ADHD symptoms. Between-group differences remained significant after controlling for background variables.

These findings are consistent with prior research demonstrating the effectiveness of psychosocial interventions in reducing ADHD symptoms and associated comorbidities, as well as in improving parental self-efficacy [27, 58, 65, 115, 126]. Overall, the intervention effect relative to the control group yielded small to moderate effect sizes. This is consistent with a recent meta-analysis [40] reporting that behavioral parent training typically yields moderate effect sizes. Nevertheless, given the low cost and scalability of the intervention, even less than moderate effects may carry meaningful practical significance.

The study findings, which indicate improvements both in parental efficacy and in the children’s symptoms following a combined parent-child intervention, allow for the proposal of several causal hypotheses: a parent-based direction, according to which it is possible that changes in parental efficacy and conduct led to changes in the child’s environmental conditions and to symptomatic improvement; a child-based direction, according to which it is possible that improvement in the child’s behavior led to improved parental efficacy; and a reciprocal direction, according to which it is possible that mutual influences between parent and child occur over time. Further research is required in order to systematically examine the mechanisms of action and the directionality of the influences between the variables [3, 83, 114].

Open-ended responses written by parents at the end of the questionnaire provide a complementary perspective. An informal analysis identifies recurring themes: a shift in understanding the child (“putting on the child’s glasses”), the attribution of positive meaning (“things we had regarded as a ‘problem’ became a tool”), and a sense of competency (“we learned to rediscover our wonderful daughter. The difficulties did not disappear, but this gives us strength and wisdom to cope”, “I feel calmer and more confident”). Alongside the parental change, there are also indications of change in the child (“there were hardly complaints about behavior”), pointing to a possible reciprocal influence. These findings are consistent with the neurodiversity perspective, which views ADHD as an expression of a mismatch between the individual and their environment, and are in line with previous research that examined an SEA-based intervention [22, 133]. Taken together, they provide preliminary evidence for the ecological validity of the current study’s findings. Further research is recommended to include systematic measurement of changes in attitudes and perceptions of stigma.

The study findings underscore the importance of tailoring intervention approaches to the specific characteristics of the target population. Accessible interventions for conservative religious communities, such as the UOJ community, are especially important given religious barriers and challenges in accessing care within the health system [10, 103, 137]. Previous studies suggest that culturally sensitive interventions should address both treatment content and culturally characteristic patterns of help-seeking behavior [35, 130]. This consideration is particularly salient in conservative religious communities, where perceptions of ADHD are influenced by religious values [10]. Accordingly, the intervention examined in this study employed conceptualizations, content, and metaphors aligned with the UOJ value system, developed in consultation with religious and educational figures from the community, and delivered through platforms accessible to this population, including audio-based spaces and filtered content [32, 50].

Cultural adaptation may explain the high participation rate in the current intervention (87%), compared to reported response rates among minority groups [151]. These findings are consistent with previous studies on the effectiveness of culturally adapted interventions [48, 84]. Moreover, although the neurodiversity approach developed in a Western context [64, 97] and its application to interventions for ADHD has been examined in the existing literature [19, 37, 122], the present study demonstrates that it can be adapted to culturally diverse communities, as reflected in the SEA approach.The intervention was delivered remotely in a digital format, enabling the simultaneous participation of a large number of families. Prior research has recommended the use of digital interventions for ADHD to address shortages in the mental health workforce [27]. The effectiveness indicators observed in the present study are consistent with the literature supporting the efficacy of online interventions for ADHD, with some evidence suggesting a particular advantage for telemedicine-based approaches [13, 125]. Potential explanations for the advantages of telemedicine include increased accessibility, which may raise referral and participation rates by eliminating the need to leave home; a greater likelihood of joint participation by both parents in sessions, potentially enhancing mutual support; and the ability to revisit recorded content that remains available beyond the live sessions.

Overall, these findings highlight the contribution of the SEA approach at both the theoretical and applied levels. The approach offers an applied framework for an affirmative intervention that integrates the medical model with the neurodiversity perspective, rather than positioning them as a dichotomy [69, 116]. Previous studies indicate that neurodivergent individuals already make use of both approaches interchangeably [79]. In the professional literature, several theoretical models for integrating the two approaches have been proposed [16, 38, 53, 62, 121, 152]. However, most of these models focus on autism or on neurodiversity in general, underscoring the need for a framework that addresses the specific case of ADHD (Brown, A. I., 2023; [68, 69]). The present study contributes to the emerging literature on the development of neurodiversity-affirmative interventions for ADHD and their adaptation to diverse populations.

### Study limitations

Despite the positive trends observed among children and parents participating in the program, several limitations should be noted. First, group allocation was not randomized and was based on the timing of parents’ enrollment in the program. However, analyses indicated no significant baseline differences between the groups. In addition, approximately 30% of the children did not have a formal medical diagnosis of ADHD; evertheless, the proportion of diagnosed children did not differ between groups. Moreover, analyses restricted to formally diagnosed children yielded comparable results, suggesting that neither diagnostic status nor diagnostic heterogeneity provides an alternative explanation for the findings.

To strengthen external validity, future studies should replicate the intervention in additional conservative populations, such as the Arab community in Israel [78]. Furthermore, the study focused on short-term outcomes and relied on parent-report measures without objective assessments or blinding of participants and researchers. Long-term follow-up studies incorporating additional informants, such as teachers or independent evaluators, are warranted. Finally, the present study examined outcome variables, including symptoms, functioning, and parental self-efficacy, rather than testing underlying mechanisms such as sense of coherence or motivation, which remain theoretical hypotheses for future research. It is important to note that SEA is proposed as a conceptual framework that operates alongside, rather than in place of, conventional medical perspectives on ADHD. Accordingly, the intervention presented this framework alongside alternative perspectives, allowing participants to choose the narrative most meaningful to them.

### Policy implications and practical recommendations

The high prevalence of ADHD, alongside a shortage of professionals, underscores the need for accessible and scalable interventions [27]. The digital format, high accessibility, and simple language of the current intervention position it as a potential direction for integration into existing systems - in education, as a platform for creating a shared language among parents, children, and staff; in healthcare, as a complementary psychosocial component to standard treatment; and in social services, as part of programs aimed at strengthening parental efficacy. These characteristics may also support the adaptation of the intervention to other conservative or religious communities. However, further research is required to examine feasibility, effectiveness, and implementation on a large scale.

### Summary & conclusion

The findings indicate that an affirmative intervention based on the SEA approach is effective in the short term in improving parent and child functioning among families with ADHD symptoms in the UOJ community. The results suggest that interventions combining multiple approaches and incorporating cultural adaptation may constitute a valuable addition to standard ADHD treatment. At the same time, future research should include randomized designs to establish causal validity, long-term follow-up to assess the stability of effects, extension to additional conservative communities, and examination of mediating mechanisms derived from the theoretical framework to clarify how the observed improvements are achieved.

## Data Availability

Due to the sensitive nature of the data and ethical restrictions, the datasets are not publicly available.

## Declarations

During the preparation of this manuscript, AI-assisted language tools was used for linguistic and stylistic assistance. The authors reviewed, edited, and modified the content as needed and take full responsibility for the final version.
